# More than “hematology”: a qualitative study on the experience of hematologists treating people with blood cancer in Greece

**DOI:** 10.1007/s00520-025-09320-0

**Published:** 2025-03-20

**Authors:** Nikolaos Vrontaras, George Koulierakis, Dimitrios Kyrou, Anargyros Kapetanakis, Aliki Xochelli, Paolo Ghia, Kostas Stamatopoulos, Christina Karamanidou, on behalf of ERIC, the European Research Initiative on Chronic Lymphocytic Leukemia (CLL)

**Affiliations:** 1 Institute of Applied Biosciences, Centre for Research and Technology Hellas, 6th Km. Charilaou – Thermi Road, P.O. Box 60361 GR, 57001 Thermi, Thessaloniki Greece; 2 Department of Public Health Policy, School of Public Health, University of West Attica, Athens, Greece; 3 Ippokrateio General Hospital of Thessaloniki, Thessaloniki, Greece; 4 Division of Experimental Oncology, IRCCS Ospedale San Raffaele, Milan, Italy; 5 Università Vita-Salute San Raffaele, Milan, Italy

**Keywords:** Hematologist, Hematological neoplasm, Neoplasm, Delivery of health care, Qualitative research

## Abstract

**Purpose:**

The present study aims to investigate the experiences of hematologists providing care to patients with hematological malignancies, whose care is pertinent to oncology.

**Methods:**

Semi-structured interviews with 30 hematologists across Greece were conducted. The interviews took place over the course of 6 months at 2020. Reflexive thematic analysis was employed for data analysis.

**Results:**

Three key themes (personal impact, organizational framework, and relating to patients) and eight subthemes were generated: (1) Hematologists were greatly affected on a personal level, as they had poor life-work balance and impacted social relationships. They experienced a great emotional toll, sometimes questioning the meaning of their work. They frequently struggled with loss, by witnessing people’s passing. Nevertheless, they reported coping better over time. (2) On an organizational level, hematologists supported each other emotionally, but only rarely had formal support in managerial or administrative recourses. They were also hindered by structural restraints, both in terms of limited psychosocial training and supportive services. (3) Hematologists’ relationship with their patients increased their job satisfaction. However, they strived to keep boundaries while balancing how close they got to their patients.

**Conclusion:**

High job demands, organizational shortcomings, and emotional challenges negatively impact their well-being and pose the risk of developing compassion fatigue or burnout. At the same time, individual resources, teamwork, and strong personal relationships emerged as crucial coping elements, providing meaning and resilience. Psychosocial training and institutional support should be offered both personally and professionally to enhance hematologists’ well-being and reduce potential turnover.

**Supplementary Information:**

The online version contains supplementary material available at 10.1007/s00520-025-09320-0.

## Introduction

Hematological malignancies are a heterogeneous group of cancer currently on the rise in the Western world [1]. Globally, the overall age-standardized incidence rate of hematological malignancies increased from 1990 to 2017, while the age-standardized disability-adjusted life-years and death rates for the same group decreased during the same period [2]. This implies that the quality of care is getting better for people with hematological malignancies, although they still face many challenges [3].

Managing the challenges of providing care for people with life-threatening hematological diseases renders the hematologist’s role quite complex. It involves assessing patients [4], selecting treatment options [5], and ensuring high medication adherence [6]. Hematologists are also required to maintain patients’ and sometimes the caregivers’ quality of life [7, 8] and manage and facilitate palliative care [9–11].

To meet the demands of their role, hematologists are expected to have various skills, such as mastery over patient communication, emotional support, information, and decision-making preferences [12–15]. They have to collaborate with patients [16] as well as with other specialists within the health care team [17]. Hematologists, similarly to any other health care providers, are also required to perceive their role clearly [18] and own a great sense of clinical adequacy [19], but at the same time also be self-aware [20]. Furthermore, they need to be addressing the intricate ethical issues that might arise, in a variety of circumstances [21].

The treatment of people with hematological malignancies within a health care setting and the multi-level complexity of their role leave hematologists dealing with many of the aforementioned challenges. This often results in increased stress, burnout, and exhaustion [22–24] and impacts their well-being [25].

Despite the high demands put on hematologists and the challenges they face, there is a scarcity of in-depth studies investigating their experiences from treating people with hematological malignancies. Those that do exist focus on specific populations, like hematologists treating pediatric patients [24], specific stages of care, like palliative care [9], or specific aspects of care, such as communication between hematologists and patients [14]. These studies, however, do not explore the overall impact of working with patients with hematological malignancies. This is in contrast to the number of equivalent studies available relating to general oncology like that of Hlubocky and colleagues [26], which are constantly updated.

The present study initially focused on the communication dynamics between hematologists and patients with chronic lymphocytic leukemia (CLL) as part of the broader CLL Patient Empowerment Program launched by the European Research Initiative on CLL (ERIC) [14]. The interview guide was developed to explore communication-related themes, including how hematologists share information, facilitate patient understanding, and support decision-making processes. However, during the interviews, many participants disclosed deeply personal reflections on their experiences of working with patients diagnosed with hematological malignancies. Studies have shown that communicating with patients especially when in the context of a bad prognosis may evoke negative and discomforting feelings for doctors affecting their overall experience [27, 28]. Therefore, these disclosures extended beyond communication challenges to encompass broader emotional and professional struggles and the psychological toll of their work.

Given this unanticipated richness of data, we opted to analyze the interviews through a different lens, examining how interactions with patients shaped the experiences and challenges of hematologists. This shift aligns with the aim of understanding the impact of working with patients with hematological malignancies while situating communication as a central, yet not exclusive, element influencing their experiences.

## Methods

### Design

A qualitative approach was adopted to allow the in-depth exploration of the individual experiences and perceptions of participants [29]. Qualitative data were gathered through semi-structured interviews with hematologists treating people with hematological malignancies in Greece, which took place over the course of 6 months at 2020. The Consolidated Criteria for Reporting Qualitative Research (COREQ) [30] were used (see Supplement A).

### Participants’ characteristics

Thirty (*n* = 30) hematologists (18 females) working in hematology departments of hospitals across Athens, Thessaloniki, Heraklion, Larisa, Serres, Alexandroupoli, and Mytilini participated in the study. Participants’ clinical experience ranged from 2 to 32 years (*m* = 14.5); four were in hematology residency training and one had retired the year before. Purposive sampling was applied [31], to identify hematologists who were specialized or involved in the treatment of people with hematological malignancies. All participants were working in public hospitals.

### Data collection

The interview guide was developed after an extensive literature review and used for all the interviews (see Appendix). It was originally designed as part of a need’s analysis for the ERIC CLL Patient Empowerment Program. It aimed to investigate communication challenges faced by hematologists in their interactions with patients diagnosed with CLL. While the interviews were structured around these communication-focused questions, participants frequently expanded their responses to include broader aspects of their professional and emotional experiences. These narratives prompted us to conduct a secondary analysis, using the same data to identify themes that reflect the hematologists’ experiences.

Taking into account recent controversies on data saturation, data collection was considered complete when the analysis of the interview data yielded a conceptual structure which was considered adequate in depth, complexity, and richness for addressing the research goals and questions.

### Procedure

Participants were recruited during a seminar for hematologist where the present study was introduced and an invitation to whomever was interested in participating was extended. The interviews were conducted by one of the researchers (CK, Health Psychologist, Ph.D., researcher, female), in a place and time chosen by the participants. The majority of the interviews took place at the Institute of Applied Biosciences, in Thessaloniki, others took place in a General Hospital in Athens with major hematological department and only in one case where participant was in a remote place of residence, interview was conducted over the phone (*n* = 1). Face-to-face interviews took place in a private room and privacy was ensured for all interviews. No field notes were made. The participants were only informed that the researcher was a health psychologist and a researcher. They were all audio-recorded and transcribed verbatim. Interview duration ranged from 20 to 60 min, with an average of 33 min. Transcripts did not return to the participants and repeated interviews were not carried out as spontaneous answers would better ascribe their experience. None of the participants refused to participate or withdrew after they had given consent.

### Reflexivity

The interviewer had already read about hematological malignancies before the interviews took place, and had also interviewed patients with hematological malignancies, so she was quite familiar with the issues that patients face. However, she had not any preunderstanding regarding the perceptions and experiences of healthcare professionals treating them.

### Ethical considerations

Upon recruitment, participants were informed about the study details (e.g., purpose, the hosting organization, procedural issues). Their rights (e.g., anonymity, confidentiality, getting informed about the findings, withdrawing from the study) were particularly stressed. All participants gave their informed consent for participation and the interview’s audio recording. The interviewer had no prior relationship with the participants. Ethical approval for this study was obtained by the Research Ethics Committee of the Institute of Applied Biosciences at CERTH (Ref. ETH.COM-20) and each participating hospital.

The interview audio recordings were stored according to the EU General Data Protection Regulation [32]. Any information that could identify the participants was anonymized at the time of transcription (e.g., participants were assigned different names). To support the participants through any arising distress, an informal debriefing and information on supportive services were offered when requested.

### Analysis

The research material was analyzed using reflexive thematic analysis [29, 33] under a critical realist approach [34].

No formal transcription method was used, as it is not necessary for thematic analysis [29]. The interviews were transcribed verbatim from audio recordings and the transcription was undertaken by CK. The thematic analysis was led by NV (Health Psychologist, M.Sc., research associate, male) with the assistance of another member of the team (DK Clinical Psychologist, M.Sc., research associate, male). The analysis was performed according to the approach and guidelines of Braun & Clark [29]. After the interviews were thoroughly read, inductive coding was performed to develop initial concepts from the data. Next, the developed codes were grouped into higher-level groups, i.e., subthemes, that encapsulated repeated patterns across the data. Then, subthemes were clustered in overarching groups that formed the “themes,” which reflected the basic organizing concepts of the data. The external heterogeneity and internal homogeneity of the developed subthemes and themes were reviewed in a multidisciplinary research team (CK, NV, DK).

Then, the most indicative transcribed interview quotes for each theme and subtheme were selected to facilitate the presentation of themes and subthemes. The selected quotes were translated from Greek to English by NV and were checked for comprehensibility by CK. Discrepancies in coding, and in the translation of quotes, were resolved by discussion until consensus was reached.

All data during the coding process were managed with Microsoft Word.

## Results

**Fig. 1:**
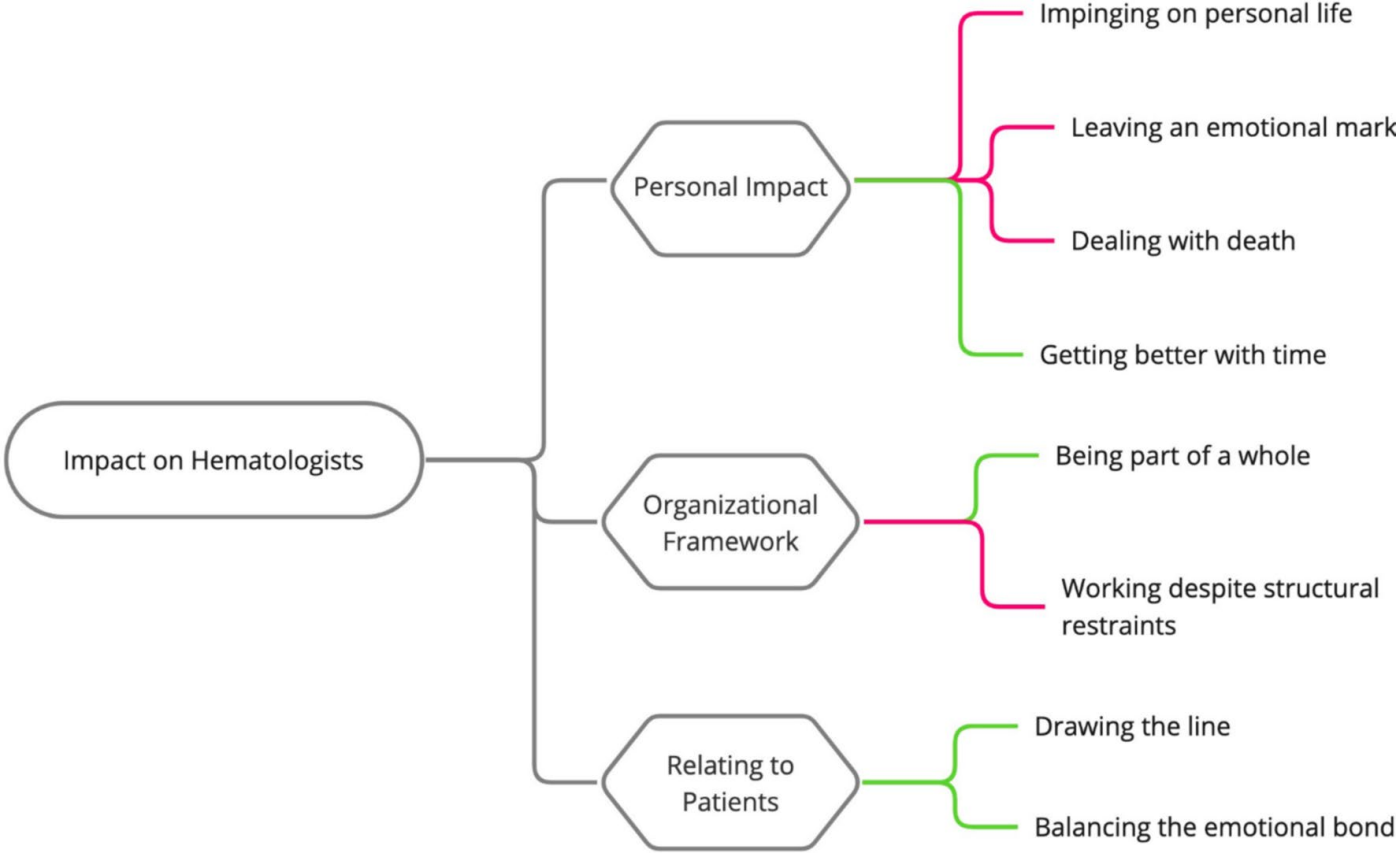
Themes and subthemes. *Note*: Red lines highlight challenges, while green lines depict facilitating factors

Exploring the impact of treating people with hematological malignancies on hematologists resulted in three key themes and eight subthemes, which are outlined in detail below, alongside indicative quotes. Themes represent the major personal impact, the organizational framework hematologists work in, and how they are relating to their patients (see Fig. 1).

### Personal impact

Hematologists described the impact on their everyday life and on their emotions, sometimes due to coming in contact with death, and how this experience changed over time.

#### Impinging on personal life

All participants described their personal life being affected by their profession. This was demonstrated by routinely returning home and continuing to work. A few described poor “*work-life balance*” as an inability to “*turn your mind off*” and because of their work’s intense nature, it was “*hard to leave it behind*.” A few reported that their sleep quality was impacted (e.g., nightmares). Many mentioned strained or more distant relationships with family or friends, as they did not have the energy, time, or willingness to engage in “*trivial conversations*.”

*I wake up in fear. … The cases are adding up, many of them aren’t doing well, do you know what I mean? … The complications are adding up, the deaths are adding up. Frequently I am tormented by [thoughts of] patients on the weekends, ‘did I do this well?’, ‘let nothing happen to them.’—Female, 7 years or experience*

#### Leaving an emotional mark

Hematologists were impacted on an emotional level by their job, resulting in a variety of negative and long-lasting effects. Some reported having developed a more fragile and sensitive mental state, making them consider accessing psychological support. Managing patients’ anxiety and fears was difficult for many, as it could gravely affect their own emotions.

*You are left feeling like you are suffocating, that you can’t manage… I have been drained out…—Female, 10 years of experience*

Many participants also had existential questions about finding meaning in their work and the everyday lives of their friends and family.

*Because you see so many people suffer, I feel you grow indifferent to your own difficulties. … you end up more “damaged” as a person… … there is no turning back to the way things were.—Male, 4 years of experience*

Some struggled with thoughts around “what if it had happened to me or my family” and how they would cope with it. Others thought their work was futile, suggesting that since they could not preserve patients’ life, there was no other way for them to help.

Many hematologists singled out a few very challenging situations in the profession that took a great emotional toll on them. One was the delivery of bad news (e.g., reporting toxicity levels, the effectiveness of the treatment, relapses, a terminal diagnosis, the unavailability of other treatments). Treating younger people was extremely stressful for many, since “they have their whole life ahead of them.”

*The emotional load is great. … Especially, if you are dealing with younger people, and you also have their family to deal with. It’s not easy. … Relapse is where things get really difficult.—Female, 31 years of experience*

One participant showed the universal nature of this by saying, “*everyone has shed tears over losing young people to a malignant disease*.”

Lastly, the withholding of information was creating internal conflicts in hematologists, making them feel uncertain or dishonest on the one hand (“*like you are lying to them, … to give them hope*”), but leaving room for patients’ hopes on the other. Participants reported struggling in front of such ethical dilemmas, which could also have legal repercussions.

#### Dealing with death

Hematologists shared their experience around people’s passing and how they coped with it. Many found it particularly difficult and tried to avoid being physically present at a patient’s death, especially when they had developed a relationship with them. Over the years, a new death would bring back memories of previous accumulated losses, leading to emotional exhaustion.

Instances they knew someone would soon pass (e.g., terminal diagnosis) were also challenging. Some struggled to understand the reasoning for offering an intense treatment that would reduce a patient’s quality of life without significantly prolonging it.

*For approximately the first nine months, I used to say that I am virtually only seeing people die. It even became my motto ‘everybody dies’. Then I asked myself, “what is it that we are even doing here?” If someone is meant to die, should I offer them three more months to live? … as one of the great philosophers put it ‘life is a series of small deaths’.—Female, Residency*

In such cases, they would also emotionally distance themselves from patients to protect themselves from the expected passing.

#### Getting better with time

Many participants talked about things getting better over time, either because they were changing, growing, and healing. This belief seemed to be widely adopted and frequently discussed among hematologists. Participants would realize and accept their boundaries while growing more confident and not questioning their decisions as much. Indicating the development of resilience, a few hematologists said that they are no longer bothered by minor, trivial things.

*Listen, you start off young and since in an end-stage disease losing young patients or especially likeable older ones gets to you. It is only with time that you develop a philosophy of life and you understand what death is and how it should be dealt with and how it could… … You find a balance there.—Male, 18 years of experience*

### Organizational framework

The healthcare system was frequently perceived as a barrier to their practice and would cause challenges, yet, working with others in a team was very supportive.

#### Being part of a whole

Hematologists praised the benefits of working with other professionals, considering it supportive and helpful. Not only would they rely on the team in decision-making, but also, they would informally engage in peer-support.

*There are usually two or three [treatment] options, each with its pros and cons. While in a clinic, the team will help you a lot with this. … We were a great team, we had the benefit of greatly appreciating and trusting each other, not being competitive with one another. … I remember this team with much affection. … I miss them even after all these years.—Female, 32 years of experience*

Nearly every participant talked about residency during which the team protected them against professional challenges (e.g., sharing responsibility for decisions).

*Since as a resident you always have the backup of a specialist, you don’t have the responsibility. … Now that I am a specialist, I clearly handle situations differently.—Female, 3 years of experience*

Hematologists recognized that working within large organizations offers resources and options, mostly related to their scientific development, sometimes in the form of managerial and administrative support, which allowed them to dedicate more time to patients (e.g., longer appointment slots) or research activities. Moreover, when hematologists reported major impact from their work, senior management facilitated the situation by rotating them into a different department (e.g., laboratory work).

*Initially, I struggled … I then changed departments … I calmed down; my family and my colleagues here helped me through it—Female, Residency*

#### Overcoming structural restraints

Hematologists also faced many structural limitations.

*It’s a given that Greek reality is making everything difficult for me. I feel it is basically an opponent, sitting on the opposite side and placing obstacles your way, either through the technical infrastructure or through the practice of one’s specialty…—Male, 8 years of experience*

Poor working conditions and lack of formal training in psychosocial skills (e.g., communicating with patients) were mentioned. In addition, a few also said that they had to learn through trial and error and through observing senior hematologists.

Additionally, participants highlighted the scarcity of psychological services in Greek hospitals. As a consequence, they alone shouldered the psychological support of their patients. Even when such services were available, sometimes patients refused a referral preferring to be supported by their hematologists, which indicates a closer relationship with them.

*It goes without saying that they would want to talk to you… not to the specialist [psychiatrist] as they are uncomfortable, they wouldn’t want to take up their time… So, you are the one listening to everything—Female, Residency*

### Relating to patients

Hematologists tried to not get too close to patients as this could possibly have an emotional impact on them. Nonetheless, they enjoyed the connection they had with their patients.

#### Drawing the line

To manage the physician-patient relationship, participants were setting boundaries for themselves and their patients.

*I want to maintain the face and standing of the physician, meaning that professionally I would not get too emotionally involved, maintain a middle ground and my professionalism. Demonstrate that I care without reaching their depth of sorrow because if I do, I will take this sorrow with me back home.—Female, Residency*

Hematologists talked about not sharing their own emotions with their patients, or interacting with them outside the hospital and avoided sharing their personal mobile number, preferring to communicate with one representative of the family. In some remote or isolated areas, this could become an issue, as they might meet in the community and the residence of the physician would be known to their patients.

#### Balancing the emotional bond

A few hematologists mentioned that getting close to patients could lead to a painful experience, in case there was a negative unexpected outcome.

*I think in this line of work, we all have lost a favorite case. Four years is a long time for you to say ‘it never happened to me’ or that ‘I may have lost people but it hasn’t affected me much’.—Female, Residency*

Nevertheless, they also mentioned the rewards of building close relationships with patients, saying “generally it is a very rewarding profession, you get back as much as you give, more than that usually.” Many enjoyed positive outcomes which brought expressions of gratitude from patients (e.g., wedding invitation, showing pictures of a new baby).

*I mean I take back plenty from these people, little things you notice as you receive their love and I consider this as a positive overview. This is what at the end of the day justifies all you do.—Female, 19 years of experience*

A few explained that they felt connected on a personal level with young people, especially when they themselves were parents. Notably, few participants emphatically spoke about putting in the effort to develop strong relationships even with people who were initially challenging to approach as worthwhile. Overall, the participants spoke warmly about enjoying practicing hematology very much.

## Discussion

This qualitative study explored the impact of treating people with hematological malignancies on hematologists, in Greece.

Initially, the study premise was to examine communication challenges, but unexpectedly, questions about communication revealed emotionally taxing experiences for the hematologists. Research has shown that physicians often feel unprepared and stressed when breaking bad news, as it conflicts with their professional goal of healing. This difficulty is particularly pronounced when discussing the termination of treatment and preparing patients for end-of-life care. Managing patients’ emotional reactions and the perception of taking away hope are among the most challenging aspects [28]. Doctors may adopt unproductive coping strategies, such as focusing on technical details while avoiding the main message [35, 36]. Unsurprisingly, effective communication remains a significant challenge for hematologists, who must navigate emotionally charged discussions, deliver difficult news, educate patients about disease management, and involve them in treatment decisions [14, 37–39]. A key finding is the emotional responses elicited even when hematologists are simply asked about their communication practices. This insight may show that difficulties in doctor-patient communication may stem from the coping mechanisms hematologists employ to shield themselves from the emotional toll of their work.

Findings indicated that treating people with hematological malignancies has a significant impact on hematologists at a personal level. Participants experienced strong emotions due to their daily exposure to loss, death, and their patients’ physical and psychological pain. Doctors in the study reported experiencing worries and intrusive thoughts, seclusion and detachment from significant others, sleep disturbances, and a loss of meaning and purpose. These symptoms have been linked to mental health issues and occupational challenges, such as burnout [31, 34]. Furthermore, many of their responses concerning how hematologists relate to their patients indicate compassion fatigue [40, 41] characterized by reduced compassion towards others. Nevertheless, hematologists also described their relationship with their patients as supportive; they enjoyed their work and found it meaningful, which relates to compassion satisfaction [40]. Notably, participants reported that the balance between compassion fatigue and compassion satisfaction shifted at different times in their careers.

Identifying and addressing the emotional burden in healthcare professionals is important, as it has been associated with reduced well-being, decreased job satisfaction, higher turnover rates, increased physical and emotional disturbances [40, 42], and, most notably, suboptimal patient care and lower patient satisfaction [43]. The emotional burden on healthcare professionals can lead to a cycle of burnout, and diminished quality of care, further compromising patient outcomes and satisfaction. Furthermore, interventions targeting compassion fatigue, such as those described by Figley [44], could serve as proactive measures if offered to hematologists, helping to enhance job satisfaction and strengthen their relationships with patients.

Another finding was the ambivalent feelings that hematologist in Greece reported about the organizational framework; however, they acknowledged the positive effect of team’s support. Compassionate team work can improve healthcare professional wellbeing, resilience, and patient care [45]. Multi-disciplinary team (MDT) meeting is a valuable tool, increasing efficiency, fostering closer working relationships, and enhancing the decision-making process [46]. Radiologists’ participation in breast cancer MDT meetings alleviated workplace isolation, enhanced their confidence, and improved patient care by sharing experiences with other colleagues [47]. Additionally, lower distress in healthcare professionals was associated with perceived organizational support [48].

However, the systematic hinderances caused by the structure of the hospital create negative feelings. Greek public hospitals and certain services are often heavily understaffed [49]. Hematologists are sometimes left without formal training, especially in psychosocial support, or adequate support while having to draw only from their own experiences, intuition, and mentors [14]. At the same time, the effectiveness of the Greek health care system lags considerably behind other EU countries in addressing treatable cancer types (e.g., breast, cervical, prostate) [50]. As the healthcare system’s performance is reciprocally linked with medical education, specific reforms to healthcare personnel’s education have been suggested [51]. These include an emphasis on primary health care education and the provision of continuous medical education by a structured framework. The above should help prepare and upskill hematologists in managing the challenges of their everyday work. The integration of multidisciplinary teams in oncology and specifically services for psychosocial support would increase quality care of cancer and at the same time improve the work environment for oncologists.

### Clinical implications

This study examines the experiences of hematologists, emphasizing findings that carry significant clinical implications for improving future practices. To manage the aforementioned challenges in clinical practice, specific educational initiatives [52], models [53], and occupational strategies like rotation [54] have been developed and implemented with varying degrees of success. Materials and training developed for oncologists could be studied for their appropriateness, adapted, and offered to hematologists treating patients with hematological malignancies by experts in psychology and healthcare communication. Support, including administrative assistance, psychosocial skills training, and access to specialized psychological services, should also be offered to manage the clinical and the emotional load mediate the impact, and reduce staff turnover.

## Strengths, limitations, and future research

The experiences of hematologists treating adults with hematological malignancies constitute an understudied area as most research focuses on the impact of oncology practice [26] and pediatric hematology [24]. Consequently, although many initiatives exist, they focus on different specialties rather than focusing on the challenges faced by hematologists in malignant hematology.

Regarding the study’s limitations, first and foremost was that the aim questions of the interview were not designed for the content that was produced. Therefore, the data collected may not fully align with the intended scope of the research, potentially limiting the depth of analysis and the applicability of the findings. However, we do feel that the findings offer meaningful contributions to understanding the subject matter and reveal important implications despite the interview design. Another limitation was that all participants were working in Greece, the findings of the present study are context-specific in several aspects. In addition, another limitation was the heterogeneity of the study’s sample as manifested in the wide range of their years of experience.

Future studies could focus on the exploration of the experiences of more specific groups of hematologists, like those of residents, and investigate specific unmet training needs. Even though initial findings on coping strategies were presented, coping could be investigated further. Overall, future research should focus on further examining the areas highlighted (e.g., personal impact, organizational framework, and relating to patients) and explore, hematologists’ work satisfaction, coping with stress, and burnout levels.

## Conclusions

To the best of our knowledge, this is the first study to offer insights into the perceptions, challenges, and experiences that hematologists adopt to deal with the impact of their work. In conclusion, it appears that working with patients with hematological malignancies in the Greek health system posed many challenges to hematologists and put them at risk for burnout or compassion fatigue. Hematologists managed to cope through the utilization of personal resources and collaborative efforts. They usually had to go beyond their formal role to provide their patients the proper care. Assistance ought to be provided on a personal and professional level to enhance their well-being, prevent turnover, and support them to continue providing high-quality care to their patients.

## Electronic supplementary material

Below is the link to the electronic supplementary material.

Supplementary file1 (DOCX 21 KB)

## Data Availability

The datasets generated and analyzed during the current study are not publicly available due to the fact that the interview transcripts are in participants’ native language, i.e., Greek, and therefore not suitable for deposit in a public repository. They can be available to anyone interested upon request to the authors of the study.

## Appendix: Interview guide

In the beginning of each interviewing session, the nature and aim of the study will be introduced:

“The purpose of this investigation is to learn more about your experience of treating patients with hematologic malignancies. Specifically, to find out about your needs and concerns around communication within the context of physician-patient relationship. We would be grateful if you could help us by sharing your thoughts and personal experience with us. If you decide to take part, the researcher will come and see you at an appropriate time and place of your choice. During this meeting, the researcher will spend a short time asking you a few questions about your experience of treating patients with hematologic malignancies. This should not take more than one hour of your time.”

Interview Guide

- Can you describe your overall experience? How would you rate it?
- What sort of contact do you have with patients? (Frequency, duration, kind, patient/ doctor’s own initiative or predetermined)
- Who provides patients with information? Would you be the main source of information? If so, how? (Face to face, communication, letter, etc.).
- What sort of information do you elicit from your patient? (Feeling, thinking, behavior, information needs, concerns). How?
- What kind of assessment do you usually perform except monitoring biomarkers? (e.g., quality of life, anxiety and depression, self-reported compliance, social support, health and illness perceptions)
- What sort of information do you communicate to your patient? How?
- How would you describe the process you follow? (Update, lecture, psycho-education, discussion etc.)
- What kind of tools do you use and if so, which? (Decision-making trees, charts, leaflets etc.)
- How you reach a decision? (Together vs. primarily yours)
- How do you inform patients about the progression of the disease? (e.g., symptoms of relapse)
- What kind of feedback do you routinely give the patient? (e.g., on the molecular response) and how?
- Which communication channels do you keep open?
- Do you involve the patient’s family?
- What kind of support do you recommend to patients ( e.g., support groups, one-to-one psychological support)?
- How do you ensure patients’ comprehension of important issues?
- Which factors do you consider important in your relationship with patients?
- Which factors do you consider as contributing to effective communication?
