# Supplementary material for: More than “hematology”: a qualitative study on the experience of hematologists treating people with blood cancer in Greece
Source: Support Care Cancer. 2025 Mar 20;33(4):307. doi: 10.1007/s00520-025-09320-0 (PMC11925966; doi:10.1007/s00520-025-09320-0)
Supplement: Supplementary file 1 — Supplementary file1 (DOCX 21 KB) [file 520_2025_9320_MOESM1_ESM.docx]

SUPPLEMENT A: COREQ 32 ITEM CHECKLIST

| No. Item | Guide questions/description | Reported on Page # |
| --- | --- | --- |
| Domain 1: Research team  and reﬂexivity |  |  |
| 1. Inter viewer/facilitator | Which author/s conducted the  interview? | 7 |
| 2. Credentials | What were the researcher’s credentials? | 7 |
| 3. Occupation | What was their occupation at the time of  the study? | 7 |
| 4. Gender | Was the researcher male or female? | 7 |
| 5. Experience and training | What experience or training did the  researcher have? | 8 |
| 6. Relationship with participants established | Was a relationship established prior to  study commencement? | 8 |
| 7. Participant knowledge  of the interviewer | What did the participants know about  the researcher? | 7 |
| 8. Interviewer  characteristics | What characteristics were reported  about the interviewer/facilitator? | 7 |
| Domain 2: study design |  |  |
| 9. Methodological  orientation and Theory | What methodological orientation was  stated to underpin the study? | 8-9 |
| 10. Sampling | How were participants selected? | 7 |
| 11. Method of approach | How were participants approached? | 7 |
| 12. Sample size | How many participants were in the  study? | 6 |
| 13. Non-­‐participation | How many people refused to participate  or dropped out? Reasons? | 7-8 |
| 14. Setting of data collection | Where was the data collected? | 6-7 |
| 15. Presence of non-­‐  participants | Was anyone else present besides the  participants and researchers? | No |
| 16. Description of sample | What are the important characteristics  of the sample? | 6 |
| 17. Interview guide | Were questions, prompts, guides  provided by the authors? | 6 |
| 18. Repeat interviews | Were repeat interviews carried out? | 7 |
| 19. Audio/visual recording | Did the research use audio or visual recording to collect the data? | 7 |
| 20. Field notes | Were ﬁeld notes made during and/or  after the interview? | 7 |

| 21. Duration | What was the duration of the interviews | 7 |
| --- | --- | --- |
| 22. Data saturation | Was data saturation discussed? | No |
| 23. Transcripts returned | Were transcripts returned to  participants for comment and/or correction? | 7 |
| Domain 3: analysis and  ﬁndings |  |  |
| 24. Number of data coders | How many data coders coded the data? | 9 |
| 25. Description of the  coding tree | Did authors provide a description of the  coding tree? | 9-10 |
| 26. Derivation of themes | Were themes identiﬁed in advance or  derived from the data? | 8-9 |
| 27. Software | What software, if applicable, was used to manage the data? | 9 |
| 28. Participant checking | Did participants provide feedback on  the ﬁndings? | No |
| 29. Quotations presented | Were participant quotations presented to illustrate the themes/ﬁndings? Was  each quotation identiﬁed? | 10-17 |
| 30. Data and ﬁndings  consistent | Was there consistency between the data  presented and the ﬁndings? | 9-17 |
| 31. Clarity of major themes | Were major themes clearly presented in the ﬁndings? | 9-17 |
| 32. Clarity of minor  themes | Is there a description of diverse cases or  discussion of minor themes? | 9-17 |
